# Cryptophthalmia, microphthalmia, oronasal malformation, and hydrocephalus in an aborted equine fetus with umbilical torsion in the state of Mato Grosso, Brazil

**DOI:** 10.1007/s11259-025-11060-9

**Published:** 2026-02-28

**Authors:** Daniel Felipe Barrantes Murillo, Gabriela Camillo, Bruna Souza Serrano, Letícia Perri Almeida Luciano, Gabriela Viana Castilho Bichara, Carlos Alberto Chaves Vás, Fernando Henrique Furlan Gouvêa, Pedro Eduardo Brandini Nespoli, Rosa Helena dos Santos Ferraz, Caroline Argenta Pescador

**Affiliations:** 1 Department of Veterinary Pathobiology, College of Veterinary Medicine, Oklahoma State University, Stillwater, OK 74078 USA; 2 Laboratory of Veterinary Pathology, College of Veterinary Medicine, Federal University of Mato Grosso, Av. Fernando Corrêa da Costa, 2367 - Boa Esperança, Cuiabá, 78060-900 MT Brazil; 3 Diagnostic Imaging Sector, College of Veterinary Medicine, Federal University of Mato Grosso, Av. Fernando Corrêa da Costa, 2367 - Boa Esperança, Cuiabá, 78060-900 MT Brazil; 4 Laboratory for Clinical Analysis of Birds and Reptiles, College of Veterinary Medicine, Federal University of Mato Grosso, Av. Fernando Corrêa da Costa, 2367 - Boa Esperança, Cuiabá, 78060-900 MT Brazil

**Keywords:** Congenital anomaly, Equine abortion, Fetal malformation, Cheiloschisis, Palatoschisis

## Abstract

Facial abnormalities and brain defects in aborted equine fetuses are rarely reported, and the causes that predispose the occurrence of this condition are difficult to identify. This work reports the morphological and tomographic findings of a case of cryptophthalmia, microphthalmia, oronasal malformation, and hydrocephalus in an aborted equine fetus with umbilical cord torsion in the state of Mato Grosso, Brazil, from a primiparous Quarter Horse female, with 7 months of gestational age. The occurrence of miscarriage was associated with compromised maternal-fetal blood flow due to umbilical cord torsion concomitant with fetal malformation. The congenital defects described in this report occurred sporadically, and it was not possible to determine the cause. The concurrent presentation of congenital malformations and umbilical torsion in equine fetuses is exceptionally rare, and this report includes the first description of the use of computer tomography for the characterization of fetal congenital malformation.

## Background

Congenital malformations are structural or functional abnormalities present at birth and derived from environmental or genetic factors that occur at different stages of embryonic or fetal development (Schild 2007). Congenital malformations affect one or multiple anatomical sites, causing abortion, stillbirth, or perinatal death (Williams 2012). Different pathogenic mechanisms contribute to the appearance of the congenital defect (Mandara et al. 2011). The most common congenital malformation in horses is the contracted foal syndrome, diagnosed in foals with severe flexor contraction and fixation of distal joints of fore and hindlimbs, scoliosis, torticollis, and maxillofacial deformity and asymmetry (Crowe and Swerczek 1985; Hong et al. 1993; Giles et al. 1993; Smith et al. 2003; Williams 2012). Congenital facial abnormalities and brain defects in aborted equine fetuses are uncommon, with a specific cause infrequently identified (Bunton 1985; Agerholm et al. 2017; Amicis et al. 2019). In a retrospective study of congenital defects in horses, 4.6% of the cases presented microphthalmia, 4.3% craniofacial deformations, 4.0% palatoschisis, and 3.0% hydrocephalus (Crowe and Swerczek 1985). The objective of this case is to describe the morphological and tomographic findings of a case of cryptophthalmia, microphthalmia, oronasal malformation, and hydrocephalus in an aborted equine fetus with umbilical torsion in the state of Mato Grosso.

## Case presentation

A 7-month-gestation aborted equine fetus and placenta, from a primiparous Quarter Horse breed mare, in a farm located in the District of Nossa Senhora da Guia, in Várzea Grande, Mato Grosso, was sent for post-mortem examination at the Veterinary Pathology Laboratory of the Federal University of Mato Grosso (UFMT), Cuiabá campus. Per submission, there were no previous records of traumatic injuries, infectious diseases, or the use of teratogenic substances administered to the mare. The attending veterinarian identified the case as a sporadic abortion due to the lack of previous abortions on the property.

**Fig. 1:**
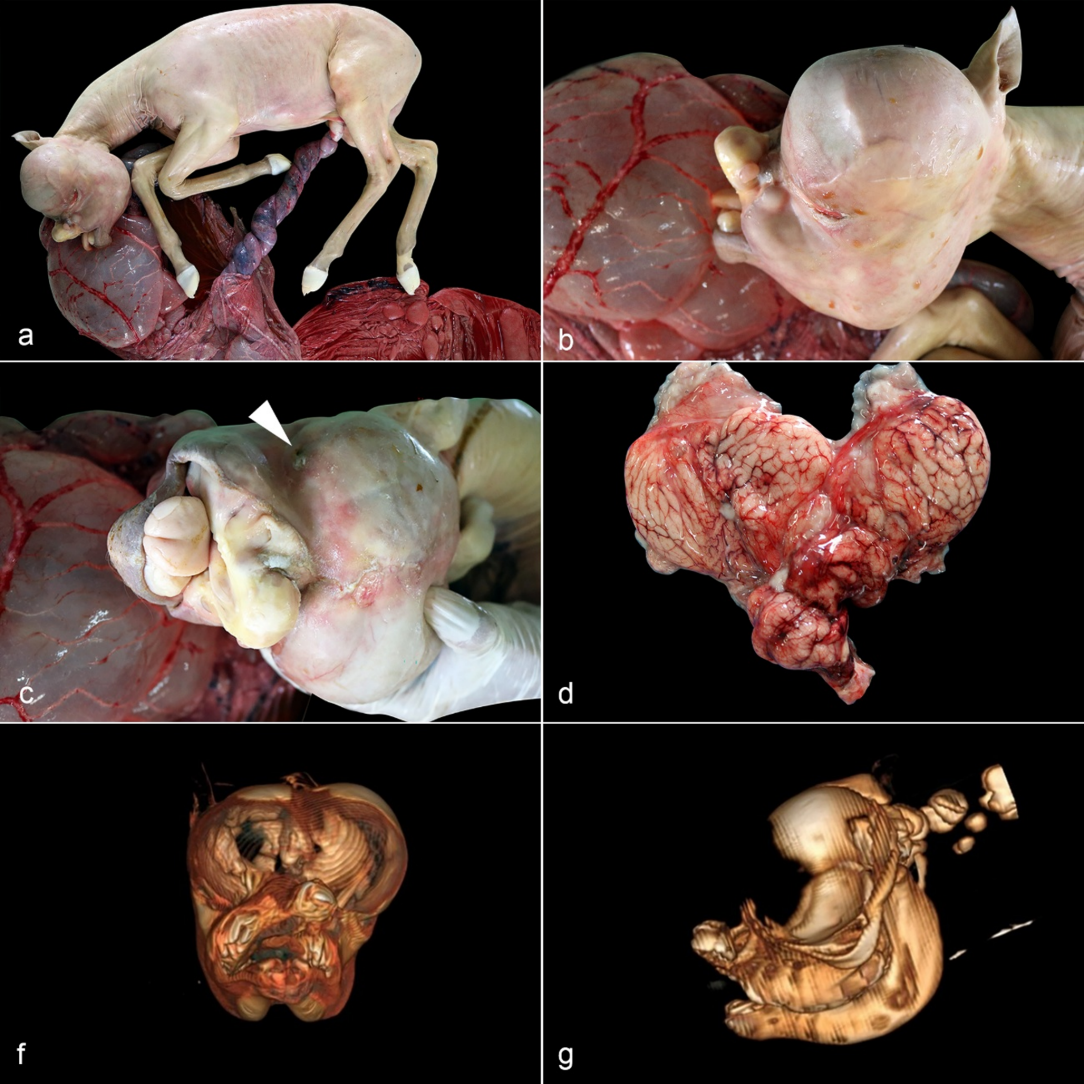
Morphological and tomographic findings of the 7-month-gestation aborted fetus. (**a**) Marked torsion of the intra-amniotic portion of the umbilical cord. (**b**) Severe craniofacial malformation and cryptophthalmia. (**c**) In cryptophthalmia, the markedly reduced ocular globe is covered by the fused eyelids (*arrowhead*). (**d**) The cerebral hemispheres (*ex-situ*) have a markedly atrophic cerebral cortex, collapsed by the markedly expanded lateral ventricles (hydrocephalus). **f-g.** Simple helical CT of the skull with a 2-channel device, Somatom Spirit^®^ - Siemens^®^, with acquisitions of 2.5 mm thickness and reconstructions in sagittal, dorsal and 3D planes. Front and left lateral view. Marked oronasal malformation with reduced nasal cavity and incomplete development of frontal and parietal bones

Macroscopically, the surface of the allantois and amnion was diffusely red, with a focally extensive dark red to black discoloration area in the cervical star region. The umbilical cord measured 70 cm and was swollen, red, and twisted on its own axis along the entire length (Fig. 1a). The crown to rump length was 63 cm (180–199 days of gestational age) (Platt 1978), and the fetal weight was 11.3 kg. The markedly pale fetus had bilateral cryptophthalmia and oronasal malformation with a wide and complete cleft in the median region of the upper lip, extending to the hard palate (cheiloschisis and palatoschisis), and a protruding tongue (Fig. 1b). The bilateral cryptophthalmia was characterized by the fusion of the eyelids, covering the markedly reduced eyes (microphthalmia) (Fig. 1c). The dorsum of the skull was asymmetric with a marked bilateral expansion caused by the mass effect of the abundant intracranial fluid. The skull was covered by a cutaneous lining, and a thin underlying muscle layer in the right occipital region. Below the skin, a thick connective tissue layer was prominent on the right side, resting directly on the pia mater, sometimes involving part of the parietal bone. The pia mater was congested, supporting a thin layer of nervous tissue that formed a cystic structure filled with a large amount of translucent yellowish fluid (hydrocephalus) (Fig. 1d). The cystic structure was formed by the bilateral dilation of the lateral ventricles. In the median region of the telencephalon, a rudimentary structure compatible with the telencephalic septum was observed. The rest of the gross examination of the fetus was unremarkable.

To better characterize the craniofacial and cerebral malformations, a helical CT of the skull with a 2-channel device, Somatom Spirit^®^ - Siemens^®^, with acquisitions of 2.5 mm thickness and reconstructions in sagittal, dorsal, and 3D planes was performed. The fetus had a distortion and an increase in volume of the cranial vault, with underdevelopment of the face, nasal, and oral cavities. Incomplete development of the frontal and parietal bones of the cranial vault was noted (Fig. 1f-g). The cerebral hemispheres were markedly enlarged, with marked dilation of the lateral ventricles (hydrocephalus). The cerebral cortex was markedly atrophied, with small peripheral tissue remaining in the ventrolateral aspect of the hemisphere. The brainstem was perceptible in the ventrocaudal portions of the cranial vault. The cerebellum had a preserved shape and was projected in the caudal direction over the foramen magnum (cerebellar herniation). The atrophied eyeballs had a reduced volume, up to 8 mm in diameter, with aphakia. The maxillary bones did not fuse dorsally and formed a single rudimentary nasal cavity, without nasal conchae, and were communicated rostrally with the oral cavity. The tongue was exposed and projected externally, and the nasopharyngeal cavity was distorted and obliterated by soft-tissue dense material. The incisor bones were displaced in the dorsal direction and to the left of the midline and presented disorganized implantation of the maxillary incisor teeth. Tympanic bullae were obliterated by abnormal soft tissue without communication to the ear canals.

Representative samples of nervous tissue, placenta, umbilical cord, lung, kidney, liver, thymus, spleen, heart, and skeletal muscle were collected and fixed in 10% buffered formalin, routinely processed for histological examination, and stained with hematoxylin and eosin (HE). Microscopically, multifocal moderate congestion was observed in the lungs, kidneys, liver, thymus, spleen, heart, skeletal muscle, placenta, and nervous tissue. The umbilical cord presented areas of edema and severe hemorrhage. Fragments of lung, liver, and stomach contents were sent to the Veterinary Microbiology Laboratory of UFMT for bacterial aerobic and anaerobic cultures. There was no bacterial growth in the samples submitted.

## Discussion

The use of CT provides a powerful tool to better describe congenital abnormalities in young horses, and it has been used for the description of a congenital maxillary malformation in a 1-month-old American Paint Horse (Tudor et al. 1999). However, there are no descriptions of CT for better identification of congenital defects in aborted equine fetuses (Agerholm et al. 2017; Roxon et al. 2023). CT provides an insightful overview of the anatomical structures of the fetus before the distortion caused by handling and postmortem examination. Through a CT scan, in this case, relevant morphological findings, including the incomplete development of frontal and parietal bones, marked hydrocephalus and microphthalmia, and aphakia of the eyes, were identified, despite the cryptopthalmia.

In Brazil, congenital malformations have been reported in buffaloes (Schild et al. 2003), small ruminants (Dantas et al. 2010) cattle (Pavarini et al. 2008; Macêdo et al. 2011) and horses (Rocha et al. 2007). This is the first case describing this alteration in the state of Mato Grosso. In general, the congenital malformations seen in animal fetuses result from several factors, such as hereditary traits, ingestion of toxic plants, teratogenic chemical substances, drugs, physical trauma, infectious agents, or nutritional deficiencies (Schild 2007); however, the definitive etiology remains unknown in the majority of equine congenital malformation cases (Crowe and Swerczek 1985; Hong et al. 1993; Giles et al. 1993; Smith et al. 2003; Laugier et al. 2011; Cantón et al. 2023).

Abortion caused by congenital abnormalities is uncommonly identified in horses and represents 2.0 to 8.5% of non-infectious abortion cases in retrospective studies (Hong et al. 1993; Smith et al. 2003; Laugier et al. 2011; Agerholm et al. 2021; Cantón et al. 2023), being more commonly identified in the second trimester and at birth (Cantón et al. 2023). Excluding the contracted foal syndrome, the most common congenital abnormality in horses (Crowe and Swerczek 1985; Hong et al. 1993; Giles et al. 1993; Smith et al. 2003), other congenital abnormalities were identified in 1.7 to 4.5% of the equine abortions in other studies (Giles et al. 1993; Smith et al. 2003). In Brazil, 1.4% of the cases of abortion in horses were attributed to congenital anomalies in a retrospective survey conducted by the Federal University of Pelotas (Marcolongo-Pereira et al. 2012). Ocular, craniofacial, and neural congenital defects described in horses include microphthalmia, anophthalmia, cyclopia, palatochisis, wry mouth, enlarged crania, anencephaly, meningoencephalocele, and hydrocephalus (Crowe and Swerczek 1985; Smith et al. 2003; Amicis et al. 2019). In our case, cryptophthalmia and bilateral microphthalmia were present. Cryptophthalmia refers to the incomplete separation of the eyelids, resulting in the skin covering the ocular globe (Landau-Prat et al. 2023). The craniofacial defects in the report described herein are similar to a previously described case (Bunton 1985), suggesting malformation of the nose and mouth, in a broad sense, as an alteration of the first pharyngeal arch, since these anatomical elements derive from this embryonic structure. This process may have started between days 18 to 21 of gestation (Hyttel and Vajta 2010). The findings in our case resemble Fraser syndrome (cryptophthalmos-syndactyly syndrome), characterized by the presence of cryptophthalmos spectrum and several craniofacial malformations, including skull ossification defects (Bouaoud et al. 2020). Fraser syndrome is attributed to the mutation of *FRAS1*, *FREM2*, and *GRIP1* genes in humans (Bouaoud et al. 2020). Unlike the case presented herein, the malformations in the central nervous system are not present in Fraser syndrome (Bouaoud et al. 2020).

In horses, the umbilical cord ranges from 35 to 85 cm, and is normally twisted, beginning from day 60 of gestation until the seventh month of gestation when fetal motility starts (Williams 2012). The umbilical cord abnormalities include edema, sacculation, torsion, or strangulation of the umbilical cord, and can result in abortion (Giles et al. 1993). Torsion of the umbilical cord is a term used to describe an excessively twisted cord with compromised irrigation and urine flow (Williams 2012). Constriction of the blood vessels, ecchymoses, thrombosis, and local edema of the vascular walls are common features of the umbilical torsion (Roxon et al. 2023). Abortions caused by umbilical cord torsion are typically seen between 5 and 8 months of gestation (Williams 2012; Cantón et al. 2023), and the reported incidence of pregnancy loss by umbilical cord abnormalities in Thoroughbreds is 1.5% after day 70 of gestation (Roach et al. 2021). The prevalence of umbilical cord abnormalities, including torsion in equine abortions, stillbirth, and perinatal death, ranges from 3.4% to 59.9% in different studies (Hong et al. 1993; Giles et al. 1993; Smith et al. 2003; Laugier et al. 2011; Agerholm et al. 2021; Cantón et al. 2023).

Concurrent congenital abnormalities and umbilical torsion in equine abortion cases are exceptionally rare. Few reports describe the occurrence of these two events in a single animal. A 304-day gestation aborted equine fetus presented with umbilical cord torsion, ventral flexion of the neck and head, and bilateral amelia of the forelimbs (Roxon et al. 2023). Another case report described a 224-day gestation equine abortion, with umbilical cord torsion and concurrent meloschisis, microphthalmia, and hydrocephalus (Agerholm et al. 2017). In a retrospective study of 57 equine abortions caused by umbilical cord torsion, only three had limb congenital malformations (Whitwell 1975). Similar to our case, the cause of abortion in these reports was attributed to the compromised umbilical blood vessels and urachus (Agerholm et al. 2017; Roxon et al. 2023). Unfortunately, the specific cause of the congenital malformations was not identified in our case.

Determining a definitive cause for the equine congenital malformation is challenging. In Thoroughbreds, congenital malformations are attributed to chromosomal abnormalities (Whitwell 1980). However, some authors discount the importance of the hereditary factors that induce congenital malformations in horses and rather speculate on an unknown teratogenic substance (Crowe and Swerczek 1985; Hong et al. 1993). The previous reports emphasize the importance of a complete clinical history and the potential exposure to teratogenic drugs at specific time points during pregnancy, without identifying a definitive cause for these abnormalities (Agerholm et al. 2017; Roxon et al. 2023). The ingestion of a teratogenic plant should be considered. In the Northeastern region of Brazil, the ingestion of *Mimosa tenuiflora* and *M. ophthalmocentra* induces craniofacial, ocular, neuronal, and limb malformations in goats (Pimentel et al. 2007; Riet-Correa et al. 2012). In Mato Grosso specifically, experimental murine models demonstrated that the ethanolic extract of *Piper glabratum* is teratogenic and interferes with the ossification (Nunes et al. 2023). In our case, the abortion was determined as sporadic, without evidence of bacterial infectious agents in aerobic and anaerobic cultures or a previous history of administered drugs, or toxic plant consumption.

## Conclusion

Congenital malformations are sporadic and infrequent causes of abortion in equine fetuses. Concurrent fetal lesions, including umbilical torsion and infectious diseases, need to be investigated. In our case, the abortion was caused by the impairment of blood flow due to the twisting of the umbilical cord. Bacterial infectious agents were ruled out through ancillary testing and histopathological evaluation. Computed tomography represented a comprehensive method to describe in detail the specific congenital abnormalities in the fetus. Unfortunately, a specific cause for these findings is seldom identified and depends on a detailed and comprehensive clinical history.

## Data Availability

The data supporting the findings of this study are available from the corresponding author upon reasonable request.
